# Phase 0 clinical trials in oncology new drug development

**DOI:** 10.4103/2229-3485.76285

**Published:** 2011

**Authors:** Umesh Chandra Gupta, Sandeep Bhatia, Amit Garg, Amit Sharma, Vaibhav Choudhary

**Affiliations:** *Clinical Research and Medical Services, Fresenius Kabi Oncology Ltd., Gurgaon, Delhi*; 1 *Medical Affairs, Novartis, Mumbai, Maharashtra*; 2 *Medical Services, Merck Serono, Mumbai, Maharashtra*

**Keywords:** Phase 0, exploratory investigational new drug, clinical trials, oncology

## Abstract

Research focus of pharmaceutical industry has expanded to a larger extent in last few decades putting many more new molecules, particularly targeted agents, for the clinical development. On the other hand, researchers are facing serious challenges due to high failure rates of new molecules in clinical studies. The United States Food and Drug Administration (FDA) in combination with academia and industry experts identified many factors responsible for failures of new molecules, and with a vision of taking traditional drug development model toward an innovative paradigm shift, issued regulatory guidance on conduct of exploratory investigational new drug (exploratory IND) studies, often called as phase 0 clinical trials, requiring reduced preclinical testing, which has special relevance to life-threatening diseases such as cancer. Phase 0 trials, utilizing much lower drug doses, provide an opportunity to explore the clinical behavior of new molecules very early in the drug development pathway, helping to identify the promising candidates and eliminating non-promising molecules, thus improving the efficiency of overall drug development with significant savings of resources. Being non-therapeutic in nature, these studies, however, pose certain ethical challenges requiring careful study designing and informed consent process. This article reviews the insights and perspectives for the feasibility, utility, planning, designing and conduct of phase 0 clinical trials, in addition to ethical issues and industrial perspective focused at oncology new drug development.

## INTRODUCTION

Since last two decades, biomedical research has progressed at a faster pace putting more and more new molecular entities (NMEs) on the drug development pathway but, on the contrary, the rate of marketing approval has shown a declining trend.[1] A typical new drug development takes 10–15 years with spending very high cost ranging from 800 million to more than one billion dollars.[2–4] About 75% of overall new drug development cost is associated with failures in early developmental stages, with <10% of investigational new drug (IND) applications reaching marketing approval decreased down from 14% in 1985,[5–7] which explains the magnitude of resources being wasted on investigating non-promising molecules. This clearly indicates that some grievances exist in the traditional drug development model, hindering the researchers to identify the true clinical characteristics of new drugs in early developmental stages. Identified responsible factors include: (a) lack of predictability of animal models in order to ascertain how the candidate drug will behave in humans;[8] (b) traditional drug development model has largely been unchanged for more than three decades with no productive innovations;[9] (c) lack of incorporation of pharmacodynamic (PD) endpoints in early stage clinical trials design to establish/confirm drug activity early in humans, particularly for molecularly targeted agents since their successful clinical development depends heavily on PD evaluati on;[10] and (d) lack of validated biomarkers that could expedite drug approval by early prediction of clinical endpoints based on biomolecular signals. Identifying the compelling need for re-evaluation of and an innovative paradigm shift in the traditional drug development model, FDA, as part of its critical path initiative, established a *Task Force on Methodology for the Development of Innovative Cancer Therapies* (MDICT) comprised of experts from National Cancer Institute (NCI), academia, industry, and FDA,[11,12] and held multiple discussions with pharmaceutical industry.[13,14] In Critical Path Report[14] issued in March 2004, FDA advocated that new tools are needed to distinguish promising candidates from those lacking promise. In January 2006, as an outcome, FDA issued guidance on Exploratory IND (ExpIND) studies.[15]

## PHASE 0 CLINICAL TRIALS

ExpIND studies, often called as phase 0 clinical trials, are conducted prior to traditional phase I dose escalation, safety and tolerability studies with very limited human exposure (<30 patients, usually 10–15 patients for a period of ≤7 days) and have no therapeutic or diagnostic potential (e.g., microdose or screening studies). These studies assess feasibility for further clinical development of a drug or biological product regulated by Center for Drug Evaluation and Research (CDER). Bridging the gap between traditional preclinical studies and clinical development, phase 0 trials provide an opportunity to assess pharmacokinetics (PK) and pharmacodynamics (PD) of new molecules early in humans with reduced preclinical testing. ExpIND approach also allows investigators to conduct phase 0 studies of closely related agents under a single IND application. Phase 0 studies have the potential of identifying promising candidates more quickly and precisely. Guidance documents by European Medicines Agency (EMEA)[16,17] and FDA[15] and work by pioneer institutions have helped in gaining recognition, acceptance and legitimacy to the conduct of phase 0 studies.

## OBJECTIVES AND TYPES OF PHASE 0 TRIALS

**Figure 1:**
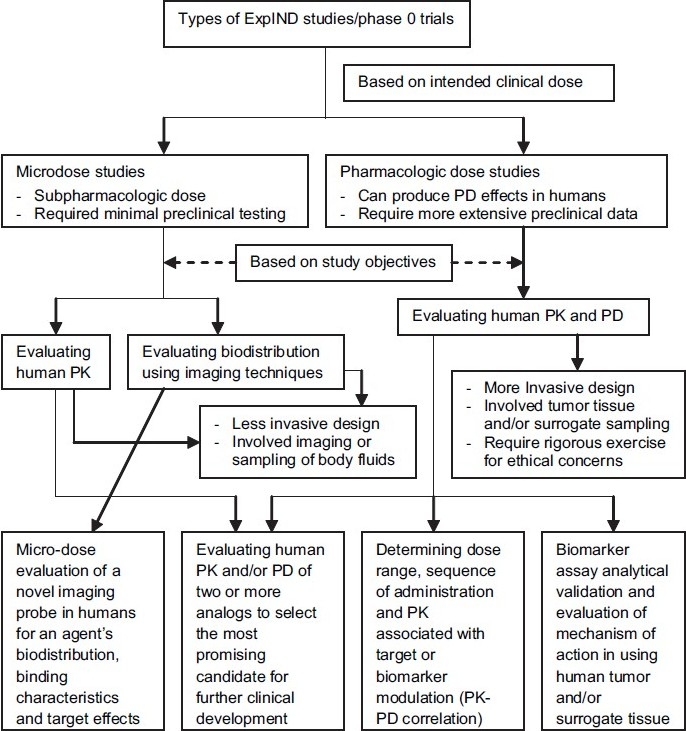
Objectives and types of phase 0 trials with their implications on phase I trials. (ExpIND, exploratory IND; PD, pharmacodynamics; PK, pharmacokinetics; MTD, maximum tolerated dose; OBD, optimal biological modifying dose)

**Figure 2:**
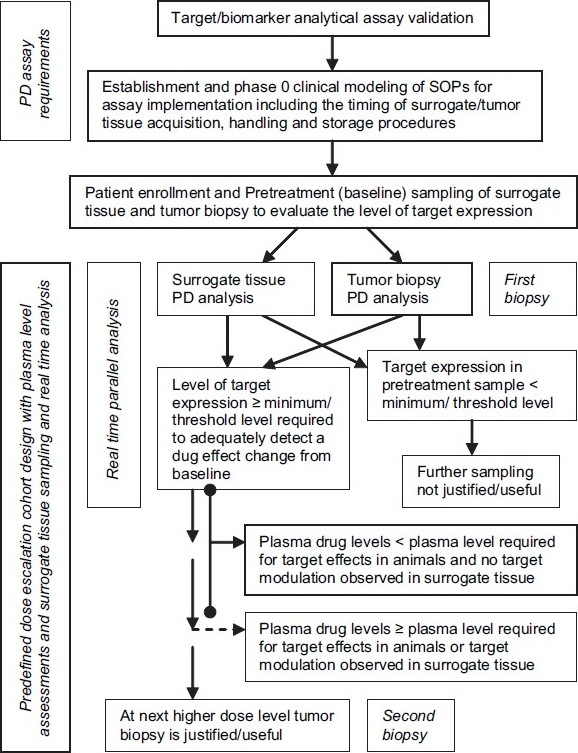
Phase 0 trial design used in ABT-888 phase 0 trial. (PD, pharmacodynamic; SOPs, standard operating procedures)

Originating from the objectives identified in ExpIND guidance, various phase 0 trial designs are possible[15,18] [Figure 1]. One type of design using pharmacologically active doses is based on the objective to demonstrate the target modulation by the drug in human tumor and/or surrogate tissue or that mechanism of action (MOA) observed in animal models can also be observed in humans. Similar approach was used in ABT-888 phase 0 trial at NCI. This trial was so designed that to minimize the invasive biopsies, post-treatment tumor biopsy was done only after achieving the plasma drug levels required to show target effects as predicated in animals or target modulation observed in surrogate tissue [Figure 2]. Second type of phase 0 trial design can be to identify the most promising candidate for further clinical development among two or more structurally similar analogues intended to act on the same target, by clinically evaluating their PK and/or PD. A third type of phase 0 trial evaluating human PK–PD correlation can be designed to determine the dose-range and sequence of administration for an agent intended to be used in combination with other agents in phase I trials. These trials could help determining in phase I trials the optimal biological modifying dose (OBD) demonstrating optimal target modulation which may be significantly lower than the maximum tolerated dose (MTD), and therefore, clinically more safer. ABT-888 phase 0 trial also used this approach and allowed researchers proceeding directly with combination phase I trials. Lastly, a phase 0 trial can also be designed for developing a novel imaging probe to evaluate clinically an agent’s biodistribution, binding characteristics and target effects. These are microdose molecular imaging studies of radiopharmaceuticals.

## DOSE SELECTION AND PRECLINICAL TESTING REQUIREMENTS

Under the ExpIND guidance, drug dose in phase 0 studies could vary from single microdose (subpharmacologic dose) to repeat pharmacologic doses. Microdose studies using microdoses (ranging from <1/100th of pharmacologically active dose in animals to not more than 100 μg or 30 nmol for protein products)[19] can be used to evaluate in humans an agent’s PK or biodistribution characteristics, with the help of novel imaging probes or technologies. Clinical starting dose of studies intended to evaluate PD is usually ≤1/50th of no observed adverse effect level (NOAEL) in sensitive species from 2-week toxicology study on mg/m^2^ basis. The maximum clinical dose would be the lowest one of the following: (i) 1/4th of NOAEL in 2-week rodent toxicology study on mg/m^2^ basis; (ii) ½ of area under curve (AUC) at NOAEL in 14-day rodent study, or the AUC in non-rodent species at NOAEL in the rodent, whichever is lower; (iii) clinical dose at which pharmacologic and/or PD response (target modulation) is observed; or (iv) clinical dose at which an adverse response is observed. Further escalation from the proposed maximum dose should be done in consultation with FDA.[20]

As the drug dose and duration of exposure in phase 0 studies are being too small to cause any potential side effects, it could be possible to initiate phase 0 studies in humans with lesser preclinical studies tailored to the objectives and nature of proposed study than the whole range of preclinical studies mandated for traditional phase I trials. Phase 0 studies intended to demonstrate an agent-related PD effects use relatively higher doses able to produce pharmacologic effects, and therefore, require relatively more extensive preclinical data as compared to microdose studies; however, it is still less extensive than what is required for traditional phase I trials as these do not aim to define MTD. Kram and Mills[20] from CDER explained the preclinical testing required for different phase 0 trials.

## DECISION MODEL FOR CANDIDATE SELECTION AND POST-PHASE 0 CLINICAL DEVELOPMENT

Before considering phase 0 trial for a new molecule, particularly PD driven study, the very first step is to evaluate whether the candidate agent is appropriate to be evaluated in phase 0 trial since every drug may not qualify as a suitable candidate. To decide on the evaluation of target/biomarker modulation, all of the following criteria apply to an ideal phase 0 candidate: (1) clinical development of the agent depends heavily on PD endpoint; (2) preclinical studies demonstrate that an antitumor effect is associated with target/biomarker modulation; (3) a wide therapeutic window is expected; (4) target/biomarker modulation is anticipated at nontoxic doses over short duration of exposure (≤7 days); and (5) target/biomarker modulation is likely to be determined with a relatively smaller sample size.[18] A wide therapeutic index and validated PD assay are required for a PD driven study; otherwise, PK driven microdose study could be a valuable option to select/eliminate a molecule.

**Figure 3:**
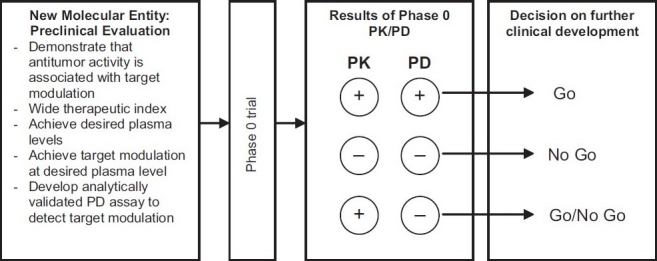
Decision model for phase 0 clinical trial and further clinical development. (PK+, desired plasma drug levels are achieved; PD+, target modulation is observed) (Adapted with permission from S. Kummar, J.H. Doroshow et al. Phase 0 clinical trials: Recommendations from the task force on methodology for the development of innovative cancer therapies. Eur J Can 2009;45:741-746.)

Post-phase 0 go/no go decisions for further clinical development rely on phase 0 trial outcomes [Figure 3]. In case of PK+ and PD–, further development will depend on the extent of preclinical evaluation and nature of PD assay used. For an enzyme inhibiting agent, for example, it is noteworthy to consider what was measured by the PD assay, whether enzyme inhibition/activity directly or as a downstream event of inhibition, for example, apoptosis. Two possibilities can be expected: (a) if enzyme inhibition was measured directly and no inhibition as an endpoint was observed, it is worth to stop further development; (b) when apoptosis, for example, as a downstream event was measured as an endpoint and no cell death was observed, it is possible that the enzyme might have been inhibited but apoptosis may require other additional events which are not a direct consequence of enzyme inhibition. In this case, use of more than one measure of PD effect could be a decision to assess the agent adequately.[19]

## PHARMACODYNAMICS IN PHASE 0 TRIALS

A PD driven phase 0 trial is intended to demonstrate the desired action of drug on its intended molecular target by measuring PD endpoint. A PD endpoint could be a quantitatively measurable variable that is capable of providing the clinically relevant and convincing evidence related to PD effect of the candidate agent. Significance of PD in phase 0 trials resides in the fact that *pharmaceutical failure* (if an agent fails to achieve adequate intratumoral levels to affect the target, measured in tumor biopsy) can be dealt with dose escalation design, however, only for drugs with wide therapeutic index; but the *pharmacologic failure* (if an agent does not affect a drug target despite achieving adequate intratumoral levels) could lead to clinical development discontinuation. Phase 0 PD data coupled with measurement of drug levels in tumor biopsy can distinguish pharmaceutical failure from pharmacologic failure. With ethical responsibility of obtaining meaningful results from biopsy specimen of each patient, relatively higher standards are required to be met as a prerequisite before the biopsies are justified in phase 0 trials.[21]

### Higher standards for laboratory assay

PD assay intended to measure the drug action must be rigorously tested for its clinical use. A valid assay should meet the performance criteria of accuracy, dynamic range, precision, reproducibility, and sensitivity. Steps to consider in PD assay development have been provided by NCI.[22]

### Successful modeling of phase 0 trial

In addition to assay validation and optimization, successful modeling of clinical procedures (for tissue collection and handling) using preclinical models is also an important prerequisite for obtaining useful assay results and assuring the assay’s clinical readiness. Importance of successful modeling with establishing optimal time window for drug administration and obtaining tissue samples is clearly evident from the past work.[23,24]

### Suitability of drug–target pair

After validation and clinical modeling of assay, it is important to evaluate the suitability of the drug-target pair as a whole for the phase 0 trial because even after having a high performance assay, tumor heterogeneity could result in a degree of sampling variability (i.e., variation in target activity or variation in PD endpoint variable) that can exceed a statistically significant change in endpoint variable expected at lower nontoxic dose levels of drug. Endpoint variable, for example, in ABT-888 phase 0 trial was the amount of poly (ADP-ribose) (PAR) measured in tissue samples, which is a product of enzymatic activity of tumor poly (ADP-ribose) polymerase (PARP), an enzyme critical for repairing damaged DNA. Small sample size is otherwise the limitation to establish a drug effect with statistical significance unless a dramatic drug effect is observed.[25] Therefore, if nontoxic dose levels of investigational agent can produce statistically significant change in the PD endpoint, the candidate drug–target pair is suitable for evaluation in phase 0 trial, for which a large magnitude of drug effect is required in case of high sampling variation in PD endpoint, whereas a modest drug effect may reach statistical significance if sampling variability is low [Table 1].

**Table 1:** Suitability of drug–target pair as a phase 0 candidate

Drug–target pairs	
Phase 0 candidates	Phase I candidates
High sampling variation in PD endpoint at baseline	If a significant difference in target activity can be achieved only at potentially toxic dose levels, either due to week drug action or high sampling variation in the PD endpoint or both
+	
Agent demonstrating large magnitude of drug effect (target modulation) that can reach/cross statistical significance at nontoxic dose levels	
Low sampling variation in PD endpoint at baseline	If target modulation by the drug is never significantly different from the baseline, either due to week drug action or high sampling variation in the PD endpoint or both
+	
Agent demonstrating modest to large magnitude of drug effect (target modulation) that can reach/cross statistical significance at nontoxic dose levels	

Therefore, to achieve significant change in PD endpoint at nontoxic dose levels, candidate drug should possess wide therapeutic index and the decision to proceed with a phase 0 rather than phase I trial depends both on the amount of variability in the target activity and the therapeutic index of the targeted agent, that is, both drug and target must qualify together, as a pair, for phase 0 trial. If an investigational agent fails to demonstrate a significant change in PD endpoint at nontoxic dose levels either due to weak drug action or high sampling variation in PD endpoint, it is not a suitable candidate and should proceed with traditional phase I trials or phase 0 trial must be designed with a more useful endpoint, that is, a different meaningful PD assay either with same or different target.

## PHARMACOKINETICS IN PHASE 0 TRIALS

About 40% of phase I failures of new drugs are thought to be due to unacceptable PK profile;[26–28] therefore, it is of significant importance to evaluate the PK of new drugs early in humans. Study is required to be designed meticulously with standardized and validated schedules of dosing and obtaining bioanalytical samples. Bioanalysis in microdose studies requires ultrasensitive and validated bioanalytical techniques able to detect very small drug quantities. PK focused phase 0 studies, being less invasive and involving only body fluids sampling or imaging, pose less ethical challenges compared to PD driven studies. Based on an agent’s PK profile, a phase 0 trial can improve go/no go decision making and can identify the most promising agent from a group of similar analogs.

For phase 0 PK evaluation, nonlinearity of PK, if exists for the molecule under investigation, may impose serious challenges for extrapolation to the PK profile at therapeutic doses, that is, whether the PK results obtained from a microdose study will, in a true sense, predict the PK results of a full-dose study required both for decision and designing of future clinical studies. This is a major concern related to PK studies because various practical aspects can deviate the PK results at microdoses.[29] Therefore, prior confirmation of validity of dose extrapolations in preclinical studies is required and expert analysis is required while interpreting and extrapolating microdose PK results.

## ACCELERATING NEW DRUG DEVELOPMENT

**Figure 4:**
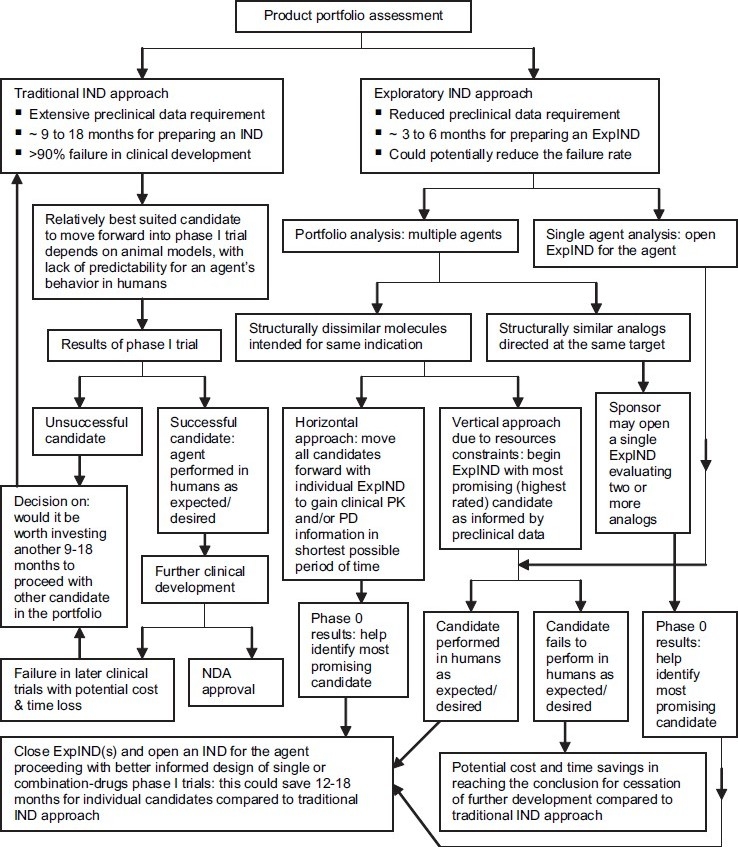
Accelerating clinical development of product portfolio by using phase 0 trials. (IND, investigational new drug application; ExpIND, exploratory IND; PK, pharmacokinetic; PD, pharmacodynamic; NDA, new drug application)

A sponsor can adopt various strategies to exploit phase 0 trials for expediting the drug development of a range of molecules[20] [Figure 4]. Further, with use of imaging technologies and radiolabeled drugs, phase 0 studies could help to identify most promising candidates with the fastest and longest lasting target effect with safest body clearance pattern.[20,30,31] Cost and time savings allowed by ExpIND are unequivocally large as compared to traditional IND for a candidate agent and more importantly much larger for evaluation of a product portfolio, which could therefore provide potential savings in long-term research and development activities.

## PHASE 0 TRIALS: STATISTICAL DESIGNS

**Figure 5:**
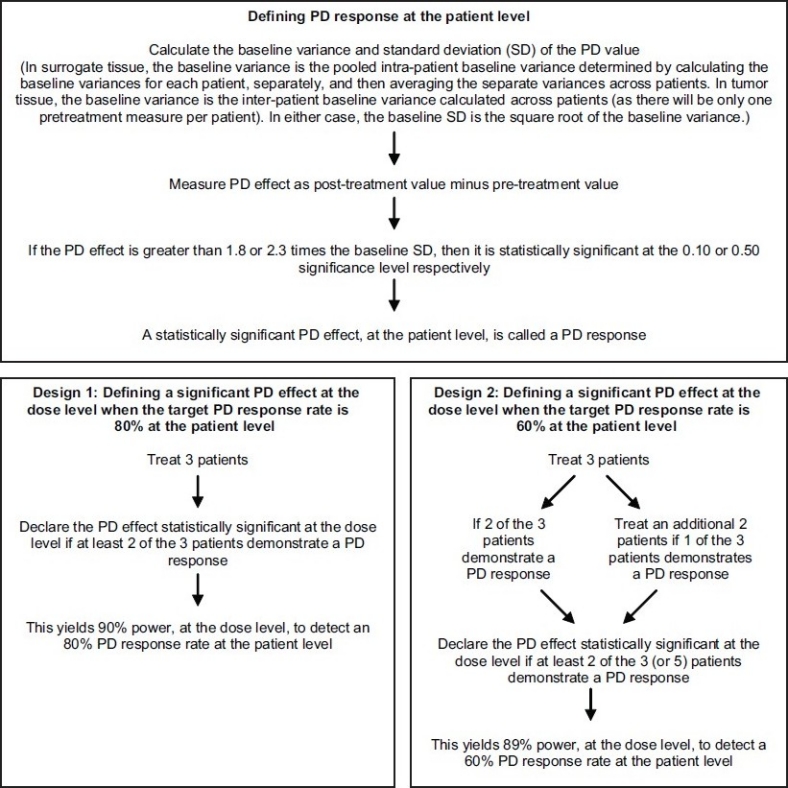
Defining PD response at the patient level and PD effect at the dose level. (Adapted with permission from Murgo AJ, Kummar S, Rubinstein L, Gutierrez M, Collins J, Kinders R, *et al*. Designing phase 0 cancer clinical trials. Clin Cancer Res 2008;14:3675-82)

Novel statistical design is required for phase 0 trials that should be valid in smaller sample size with required power and due consideration of intra- and inter-subject variability of target activity.[32] Phase 0 studies can be designed to detect a statistically significant PD response (treatment-related desired change in PD variable from the baseline value) both at individual patient level and dose levels[18] [Figure 5]. PD endpoint, ideally, is measured both in surrogate and tumor tissue. For example, in ABT-888 phase 0 trial, peripheral blood mononuclear cells (PBMCs) were used as surrogate tissue. However, multiple pretreatment and post-treatment surrogate tissue sampling can be done to measure intra-patient variability as well as duration of target modulation, respectively; but invasive tumor biopsies, for ethical reasons, are often limited to one pretreatment and one post-treatment sampling and are therefore unable to measure intra-patient variability, which makes PD response determination in tumor tissue more difficult because of often much greater inter-patient variability than intra-patient variability. As multiple surrogate tissue sampling is possible, one pretreatment and one post-treatment surrogate tissue sample should be acquired roughly at the same time of obtaining tumor tissue sample to explore the correlation between these two PD endpoints and a uniform post-treatment primary endpoint time for later studies. In design 1 [Figure 5], a significant PD response at dose level can be defined requiring only three patients per dose level with 90% power sufficient to yield 80% response at patient level. Design 2 can determine a significant PD response at dose level requiring three to five patients per dose level with 89% power targeting 60% PD response at patient level. These designs could be adapted for evaluation of two or more analogues/dosing regimens by evaluating PD response separately for each analogue/regimen which could then be compared with more standard methods.

## ABT-888 PHASE 0 TRIAL: EXPERIENCE AT NCI

Oncology’s first phase 0 trial of anticancer drug ABT-888, a PARP inhibitor, at NCI,[33] with the objective to determine PARP inhibiting dose-range and PK was successfully completed, involving only 13 patients, and the trial objectives were met in just 5 months before initiation of any planned studies.[34] Based on phase 0 results, FDA allowed investigators to begin directly with phase I combination drug studies, of better informed design incorporating PD endpoints with efficacious but expectedly more safer starting dose based on PK–PD correlations established in phase 0, bypassing the single-drug phase I studies typically required before combination studies. Researchers claimed that about 12–18 months were saved by better informing the design of subsequent trials and beginning with higher dose than they otherwise would have.[32]

## ETHICAL ISSUES IN CONDUCTING PHASE 0 TRIALS

Being non-therapeutic in nature, ethical concerns have been raised pertaining to conduct of phase 0 trials, including no direct benefit to patients, delayed participation in other trials and invasive biopsy procedures. Comprehensive analysis of ethical issues, however, did not disclose any issue making these trials inherently impossible,[35] and these can be dealt with careful strategies focusing on informed consent process and study design. Patients should be carefully informed of no personal benefit and their understanding is required to be documented.[36] Patients who have had prior participation in other research trials can better understand the element of research.[37] Risks of delayed participation in other therapeutic trials due to participation in phase 0 trial can be balanced with short duration of exposure (≤7 days) and washout period (≤2 weeks). For each patient, a defined plan is required for overall clinical care integrated with phase 0 participation and if the drug in phase 0 trial proved to be beneficial, phase 0 participants should be allowed to participate in later phase studies.[19,38] Patients requiring immediate medical care should be excluded.[18,25]

Using relatively much lower doses with greater safety margin, the probability of adverse effects is expected to be much lower; however, close monitoring and adequate medical care similar to other clinical trials is required.[25] Recently, a methodology is proposed involving the patients, with advanced or metastatic disease, who are waiting for surgery so that post-treatment biopsies can be merged with typical surgical procedure.[39]

## INDUSTRIAL PERSPECTIVE

Phase 0 trials have potential for improving the efficiency of new drug development. Inclusion of phase 0 studies in drug development process is still questionable since these do not replace traditional phase I trials[38] and do not provide any evidence for clinical efficacy and safety; however, industry is increasingly accepting the phase 0 concept to identify the promising agents, with many pharmaceutical giants becoming pioneers in this arena.[40] Phase 0 studies also open an opportunity for research based smaller biotech companies by early demonstration of proof of principle for speedy development of own molecules or to attract investors for further clinical development. PK driven microdose studies requiring relatively lesser preclinical testing and no PD assay seem more lucrative. Cost involved in typical toxicology and phase I studies for a new drug is estimated to be 4–6 million dollars;[41] therefore, the cost saved, even after considering the phase 0 cost, would be significant by eliminating the poor molecules in addition to even larger time and opportunity cost. Phase 0 trials may have several potential benefits. However, they have certain limitations too [Table 2]. Maximum benefit out of phase 0 trials could be realized by identifying the most promising candidate or relatively promising candidates, establishing PK–PD correlation for the candidate, use of phase 0 results in go/no-go decision making, logical selection of combination regimens, and design of further clinical trials. As phase 0 trials allow assessing human PK and PD very early in the development process, it is hoped that these can be an effective addition to drug development armamentarium.[42] It is advocated to start early discussions with FDA to take the full advantage of phase 0 studies and to avoid any faulty guesswork in ExpIND preparation.

**Table 2:** Benefits and limitations of phase 0 trials

Benefits
Explore clinical characteristics of a candidate agent with very less number of patients in a short duration of time Require less number of patients Could improve success rate of overall new drug development Guide go/no go decisions for subsequent clinical development Provide better approximation of active and safe starting dose for phase I trials Could expedite clinical development bypassing single-drug phase I studies, better informed designs of later clinical studies, and use of biomarkers Serve as candidate selection tool by distinguish most promising candidate from a set of analogs By early elimination of non-promising molecules could save valuable patient resources; save cost, time and resources Shift resource utilization toward promising candidates Require lesser preclinical testing data than what is mandated for traditional phase I studies Closely related agents can be evaluated under single ExpIND Provide opportunity to develop potential biomarkers

**Limitations**

Nonlinear PK, if exists, can pose problems for dose extrapolations False negative results can lead to discontinuation of promising candidates Every drug may not be a suitable candidate for phase 0 trials
